# Sensitivity Profile of Fosfomycin, Nitrofurantoin, and Co-trimoxazole Against Uropathogens Isolated From UTI Cases in a Secondary Care Center, KSA

**DOI:** 10.7759/cureus.53999

**Published:** 2024-02-11

**Authors:** Syed Yousaf Kazmi, Kauser Fathima, Nazia Khan, Syeda Nazia Kulsum, Ali Faraz

**Affiliations:** 1 Basic Sciences, Majmaah University, AlMajmaah, SAU; 2 Pathology and Laboratory Medicine, Tumair General Hospital, Tumair, SAU

**Keywords:** uropathogens, uti, gram-negative, fosfomycin, escherichia coli, antibacterial, antibacterial resistance

## Abstract

Background

Fosfomycin, nitrofurantoin, and co-trimoxazole are cheap and effective first-line oral antimicrobials in cases of uncomplicated cystitis in males and non-pregnant females. Fosfomycin and nitrofurantoin are called urinary antiseptics because these two drugs are primarily excreted in the kidney and concentrated in the urine without systemic effect. The present study was designed to evaluate the in vitro activities of fosfomycin, nitrofurantoin, and co-trimoxazole against uropathogens isolated at King Khalid Hospital Al-Majmaah, KSA.

Methods

The study was conducted at the King Khalid Hospital Al Majmaah, KSA, from September 1, 2021, until February 28, 2022.

The patients' urine samples were inoculated on the Cystein Lactose Electrolytes Deficient (CLED) medium, and uropathogens were isolated. The organisms' identification and sensitivity testing against cotrimoxazole, fosfomycin, and nitrofurantoin was conducted using a Microscan automated analyzer, the MicroScan WalkAway Beckman Coulter, Sacramento, CA, USA.

Results

The study comprised non-repeat 137 patients who were either admitted to the hospital or treated as outpatients, yielding a total of 147 isolates. Nitrofurantoin showed a lower resistance rate, around 20% (n = 29), followed by fosfomycin at 23% (n = 34). The resistance rate of cotrimoxazole was 43% (n = 63). Overall, nitrofurantoin and fosfomycin showed relatively lower resistance against all isolates.

Conclusions

Being cheap and effective, we propose that fosfomycin and nitrofurantoin be used as first-line treatments in patients presenting with uncomplicated UTIs.

## Introduction

Urinary tract infections (UTIs) are one of the most common causes of hospital visits by patients. It is estimated that around 50% of women have experienced at least one UTI episode in their lifetime [1]. Urinary tract infections are broadly classified as community-acquired, hospital-acquired, uncomplicated, and complicated [2]. This has significant implications for managing the disease because community-acquired UTI bacteriology differs significantly from hospital-acquired UTI. *Escherichia coli *remains the most common isolated pathogen in community-acquired UTIs, which accounts for around 80%, while it causes around 65% of hospital-acquired UTIs [3].

The lower UTI, commonly called cystitis, may result in ascending pyelonephritis, septicemia, premature delivery, or low-birth-weight newborns. Therefore, it is recommended to treat UTI empirically as an outdoor patient and send urine for culture and sensitivity testing for later antimicrobial adjustment if the clinical condition does not improve. However, no single guideline can be effectively followed universally due to differences in the sensitivity profile of the local prevalence of circulating uropathogens. Due to the emergence of extended-spectrum beta-lactamase (ESBL) and carbapenemase production among Enterobacteriaceae, there is always a need for frequent revisiting of local data about the sensitivity profile of common uropathogens circulating in the community as well as in hospital settings. This helps guide the clinician in selecting the best possible antimicrobials for the empiric treatment of UTIs.

Fosfomycin, nitrofurantoin, and co-trimoxazole are cheap and effective oral urinary antiseptics with relatively lower levels of resistance against common uropathogens. A study stretching over four years from 2015 to 2019 in India showed that fosfomycin had the least overall resistance against uropathogens, followed by nitrofurantoin [4]. Many local studies in KSA also point out the low resistance rates of urinary antiseptics in uropathogens. For example, in a study conducted in 2015-16 in Riyadh, KSA, the antimicrobial resistance of nitrofurantoin among the two commonest isolated uropathogens, namely, *E. coli* and Klebsiella (*K. pneumoniae* was shown to be the lowest at 17% [5]. Another study conducted in 2013 at Armed Forces Hospital Southern Region (AFHSR), Khamis Mushayt, Saudi Arabia, showed nitrofurantoin had a resistance rate of 25% & 30% against E. coli and other Gram-negative bacteria, respectively [6]. Another recent study conducted in 2017 at Prince Mutaib bin Abdulaziz Hospital, Aljouf Region, Saudi Arabia, showed a resistance rate for fosfomycin and nitrofurantoin of 24.6% and 42.9%, respectively, against common Gram-negative uropathogens [7].

There is a shortage of local data regarding the antimicrobial profile of first line drugs for treating urinary tract infections in KSA. The present study was conducted to determine the spectrum of uropathogens and the sensitivity profile of nitrofurantoin, fosfomycin, and co-trimoxazole against common urinary isolates in patients with urinary tract infection at King Khalid Hospital, Al Majmaah, KSA.

## Materials and methods

Study design

It was a cross-sectional study.

Study setting

The study was conducted at King Khalid Hospital, AlMajmaah, KSA, which is a 200-bed secondary care hospital catering to the entire Majmaah Governorate with a population of approximately 97,000.

Target population

The study participants included patients of all ages and genders who reported to King Khalid Hospital, AlMajmaah, with a clinical diagnosis of UTI.

Inclusion and exclusion criteria

Patients of all ages and either gender who presented with symptoms of dysuria, urinary frequency, and positive urine culture in a significant count were included in the study, whereas cases with a suspected upper UTI (pyelonephritis) or genital or sexually transmitted infection (e.g., vaginal discharge) were excluded.

Duration of the study

The study was of a six-month duration, starting from September 1, 2021, until February 28, 2022.

Sampling

The complete enumeration technique was used for data collection. In this, all patients reporting or diagnosed with acute cystitis were included based on inclusion and exclusion criteria.

Data collection

The general physicians at Primary Health Centers or Consultants at King Khalid Hospital clinically diagnosed the patients as having cystitis based on their history, physical findings, or urine dipstick results. The urine samples of these patients were collected in a sterile, wide-mouthed plastic container, and the procedure for the mid-stream, clean catch sample was explained to the patient by the trained staff. The samples were ensured to reach the microbiology lab within 15 minutes of collection. The duty technician at the microbiology lab inoculated the urine sample on the Cystine Lactose Electrolytes Deficient (CLED) medium using a sterilized plastic disposable loop with a urine sample holding capacity of 1 µL. The CLED plates were then incubated at 35 ˚C aerobically for 24 hours overnight. The next day, more than 100 colonies from the sample of an uncatheterized patient or 10 colonies from catheterized patients were considered significant, corresponding to 10^5^ and 10^4^ CFU/ml of urine sample, respectively [8]. The colonies on CLED agar were identified by Gram staining, biochemical tests, and serology (Lancefield grouping for Streptococci, and clumping factor latex agglutination test for Staph. aureus) when required. The susceptibility testing was carried out using a Microscan automated analyzer, the MicroScan WalkAway (Beckman Coulter, Sacramento, CA, USA) which estimates minimum inhibitory concentrations (MICs) of microbes utilizing the broth microdilution method (BMD). The organism’s identity was finalized by the Microscan biochemical panels, and MICs were noted down for each antimicrobial.

The patients’ sociodemographic data were collected from the hospital management system, including age, gender, hospitalization or outdoor management, history of antimicrobial intake, co-morbid condition, etc.

Data analysis

Data were retrieved from a Microsoft Excel file and analyzed using IBM SPSS Statistics 24 (IBM Corp., Armonk, NY, USA). The mean and SD were estimated for quantitative variables. Frequencies and percentages were utilized for qualitative variables. 

Ethical considerations

Ethical approval of the study was sought from the IRB (Central IRB log No. 21-88 E). All data were kept confidential and utilized only for this study.

## Results

The study included 137 patients who had been admitted or treated as outpatients and a total of 147 isolates were retrieved. Ten urine samples out of these 137 patients exhibited clinically significant growth of two uropathogenic microorganisms. The gender distribution showed that the majority of the patients were females (n = 95). Regarding the age group, more than half of the patients (n = 74) belonged to the age group of 15-64 years, followed by patients above 65 years of age (n = 41). The state of admission showed that 63 patients were admitted to the hospital while 74 patients were treated as outpatients. Only 17 patients were catheterized, and the majority of patients (n = 131) had no history of antibiotic intake. The predominant microorganisms included *E. coli* (n = 59), followed by *K. pneumoniae *(n = 25), and *E. faecalis* (n = 21). Table 1 presents a summary of the parameters of study participants and the isolates in a hospital setting.

**Table 1:** Summary of parameters of study participants and isolates

Parameter		Value
Total patients		137
Total isolates		147
Gender		
	Male	42
	Female	95
Age group (in years)		
	1-14	22 (16.06%)
	15-64	74 (54.01%)
	>64	41 (29.93%)
State of admission		
	Admitted	63
	Outdoor	74
Status of catheterization		
	Catheterized	17
	Non-catheterized	120
History of antibiotic intake		
	No	131
	Yes	6
Organisms isolated		
	E. coli	59
	K. pneumoniae	25
	E. fecalis	21
	Enterobacter cloacae	5
	Proteus mirabilis	5
	Candida spp	5
	Others	27

**Figure 1:**
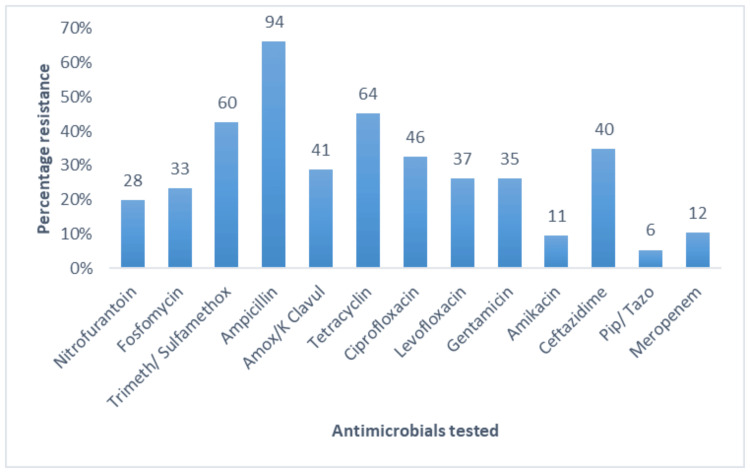
Showing antimicrobial resistance among uropathogens The number on the top of the bars signifies the number of isolates showing resistance to the antimicrobial tested. Amox/K Clavul, Amoxicillin potassium clavulanate; Pip/Tazo, Piperacillin tazobactam

Figure 1 shows that nitrofurantoin showed a resistance rate of 20% among all the antimicrobials tested, only after amikacin at 10% and piperacillin/tazobactam at 5%. In contrast, ampicillin had the highest resistance rate of 66%, followed by tetracycline (45%) and trimethoprim/sulfamethoxazole (43%). Among the fluoroquinolones, levofloxacin had a lower resistance rate than ciprofloxacin (26% vs. 33%). The resistance rate of fosfomycin, a relatively new antibiotic, was 23%.

The aminoglycoside gentamicin had a resistance rate of 26%. Among the cephalosporins, ceftazidime had a resistance rate of 35%. Meropenem, a carbapenem antibiotic, showed 10% resistance among uropathogens.

**Figure 2:**
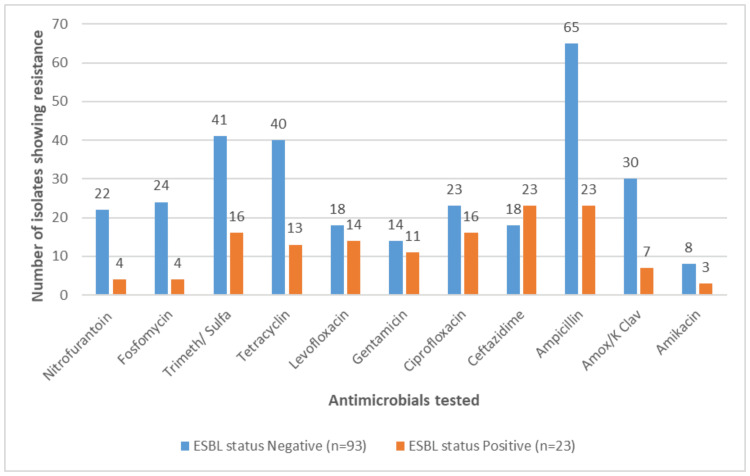
Showing the antimicrobial resistance rates among ESBL-producing and ESBL-non-producing Enterobacteriaceae causing UTIs ESBL, Extended-spectrum beta-lactamase; Trimeth/Sulfa, Trimethoprim/sulfamethoxazole; Amox/K Clav, Amoxicillin/clavulanate potassium

In Figure 2, it is shown that among non-ESBL isolates, the most effective antimicrobials were amikacin and gentamicin (resistance rates of 9% and 15%, respectively) while nitrofurantoin, ciprofloxacin, and fosfomycin were the best oral antimicrobials, with resistance rates of 24%, 25%, and 26%, respectively. In contrast, the highest resistance rates were observed for ampicillin and trimethoprim/sulfamethoxazole, with 70% and 44% resistance, respectively.

Among the ESBL-positive isolates, the most effective antimicrobials were amikacin, nitrofurantoin/fosfomycin, followed by amoxicillin/clavulanate, with a resistance rate of 13.04%, 17.39%, and 30.43%, respectively.

In contrast, the highest resistance rates were observed for ampicillin and ceftazidime, with resistance rates of 100% each. Apart from the above antimicrobials mentioned, the resistance rates for other antimicrobials varied between 30.43% and 69.57% among the ESBL-positive isolates. Table 2 summarizes the resistance patterns of common antimicrobials against major isolates in the study.

**Table 2:** Summary of resistance of common antimicrobials against major isolates in the study Amox/K Clavul, Amoxicillin clavulanate potassium; Pip/Tazo, Piperacillin and tazobactam

Antibiotics	E. coli	K. pneumoniae	Proteus mirabilis	Enterococcus fecalis/faecium
	n (%)	n (%)	n (%)	n (%)
Nitrofurantoin	3 (5.08%)	6 (24.00%)	4 (80.00%)	2 (8.70%)
Fosfomycin	5 (8.47%)	5 (20.00%)	2 (40.00%)	5 (21.74%)
Trimeth/Sulfamethox	33 (55.93%)	9 (36.00%)	3 (60.00%)	3 (13.04%)
Ampicillin	38 (64.41%)	25 (100.00%)	4 (80.00%)	5 (21.74%)
Amox/K Clavul	10 (16.95%)	4 (16.00%)	4 (80.00%)	4 (17.39%)
Tetracyclin	30 (50.85%)	7 (28.00%)	5 (100.00%)	9 (39.13%)
Ciprofloxacin	25 (42.37%)	5 (20.00%)	4 (80.00%)	7 (30.43%)
Levofloxacin	23 (38.98%)	4 (16.00%)	2 (40.00%)	5 (21.74%)
Gentamicin	12 (20.34%)	4 (16.00%)	3 (60.00%)	Intrinsic resistant
Amikacin	5 (8.47%)	3 (12.00%)	1 (20.00%)	Intrinsic resistant
Ceftazidime	20(33.90%)	8(32.00%)	3(60.00%)	Intrinsic resistant
Pip/Tazo	2 (3.4%)	3 (12.00%)	1 (20.00%)	Not tested
Meropenem	3 (5.08%)	3 (12.00%)	3 (60.00%)	Not tested
Total isolate	59	25	5	23

Considering individual antimicrobial resistance patterns among four major isolates in our study (*E. coli, K. pneumoniae, Proteus mirabilis, *and *Enterococcus faecalis/faecium*), it is evident that the highest resistance among *E. coli *was observed against ampicillin (64.41%), followed by trimethoprim/sulfamethoxazole (55.93%). *K. pneumoniae* showed complete resistance against ampicillin (intrinsically resistant) while the highest resistance was observed against trimethoprim/sulfamethoxazole (36%). All five isolates of *Proteus mirabilis *were resistant to tetracycline, and the highest resistance was observed against ampicillin (80%). Nitrofurantoin was found to be the most effective urinary antiseptic against *Enterococcus faecalis/faecium* with only two isolates (8.7%) that showed resistance, followed by trimethoprim/ sulfamethoxazole with only three isolates (13.04%) showing resistance out of 23 isolates. Overall, nitrofurantoin and fosfomycin showed relatively lower resistance against all isolates.

## Discussion

The effectiveness of antibiotics used in clinical settings has decreased due to the continued global increase of antimicrobial resistance among bacteria that cause urinary tract infections. Thus, adapting appropriate antibiotic dosage guidelines based on regional monitoring programs (surveillance) for resistant microorganisms is essential. The current research identified the incidence of uropathogens linked to UTIs and their antibiotic resistance profiles against frequently prescribed antibiotics during a six-month duration in AlMajmaah, Saudi Arabia.

Similar to earlier studies, the majority of the 137 participants in our research were females [9,10]. This is not unusual and is likely attributed to the anatomy of the female genitourinary system, particularly the shorter urethra and the close proximity of the urethral opening to the anus in females.

Our study results showed that the majority of patients were adults (n = 74, 54%), which is consistent with a study conducted in 2019 in KSA [11]. Contrarily, another study found a rise in UTI incidence among older females (>50 years old) [12], and this discrepancy in age groups might be the result of variations in the study cohort since some of the data received for this study were gathered from pregnant females.

Enterobacteriaceae were shown to be the most common family of bacteria recovered from UTI patients in our study. This result is consistent with earlier studies conducted in several countries, including Saudi Arabia [13,14], where *E. coli* was found to be the most commonly isolated bacterium for UTIs, followed by *K. pneumoniae*. In our study, Gram-positive cocci, such as *E. faecalis*, accounted for nearly a third of UTI cases. A similar pattern was observed in the southern region of Saudi Arabia (Al-Baha), where strains of these species were found among the UTI-causing bacteria [15].

Among all the uropathogens tested in our study, nitrofurantoin is among the antimicrobials showing a low resistance rate (20%), followed by amikacin at 10% and piperacillin/tazobactam at 5%. In contrast, ampicillin had the highest resistance rate at 66%, followed by tetracycline at 45% and trimethoprim/sulfamethoxazole at 43%. The resistance rate of fosfomycin, a relatively new antimicrobial chemical, was 23%. Among the fluoroquinolones, levofloxacin had a lower resistance rate than ciprofloxacin at 26% and 33%, respectively. The high resistance rate in ciprofloxacin in our study is in line with the findings in a study carried out in Kaiser Permanente WA, Seatle, USA, which stated that the ciprofloxacin-resistant UTI-causing strains of *E. coli* increased from 14.2% to 19.8% between 2015 and 2021 [16]. Comparable research conducted in Saudi Arabia revealed that bacteria were highly resistant to several routinely used antimicrobials, including ampicillin, ciprofloxacin, trimethoprim-sulfamethoxazole, and third-generation cephalosporins [17].

Current antimicrobial guidelines by the Infectious Disease Society of America (IDSA) recommend the use of nitrofurantoin, fosfomycin, fluoroquinolones, and beta-lactam agents for the treatment of acute, uncomplicated cystitis in non-pregnant adult female [18]. These guidelines recommend including trimethoprim-sulfamethoxazole in the list of empiric first-line antimicrobials for uncomplicated cystitis if the prevalence of resistance in *E. coli* is less than 20%. Our study demonstrated that the overall resistance of trimethoprim-sulfamethoxazole was 43%, which precludes its use as a first-line empiric oral antimicrobials in patients presenting with cystitis in the Majmaah region.

In a case-control study conducted in 2018 on a large sample size of 49,779 UTI cases in the Aseer region of KSA, the sensitivity profile showed that fosfomycin, nitrofurantoin, and co-amoxiclav were the best oral antimicrobials against uropathogens while co-trimoxazole showed higher resistance rate of 50% [19]. Our findings are also consistent with this study where nitrofurantoin, ciprofloxacin, and fosfomycin were the best oral agents against UTI-causing bacteria while co-cotrimoxazole showed a resistance rate of 43% overall and 41% in non-ESBL Enterobacteriaceae (Figures 1, 2).

ESBL-producing strains are associated with hospital-acquired UTIs and increased mortality. Given that the majority of the samples were from outpatients, this may explain the comparatively low number of ESBL-producing bacteria that were found in the current study (23 ESBL producers as compared to 93 non-ESBL producers). As our study points out, the best oral antimicrobials against ESBL-producing strains of Enterobacteriaceae were nitrofurantoin and fosfomycin while co-trimoxazole showed higher resistance (Figure 2). These findings are supported by a paper from Bitsori et al. in 2019 that highlighted that fosfomycin and nitrofurantoin continued to remain active against ESBL-producing strains of Enterobacteriaceae that cause UTI [20].

The current recommendations for treating uncomplicated urinary tract infections in adult male and adult non-pregnant female patients do not necessitate urine culture and sensitivity testing [18]. In most parts of the world, nitrofurantoin, fosfomycin, and trimethoprim-sulfamethoxazole are first-line treatments while ciprofloxacin and beta-lactam drugs are often prescribed as second-line drugs. In our study, trimethoprim-sulfamethoxazole and ciprofloxacin have high resistance rates overall and in multi-drug ESBL-producing strains of Enterobacteriaceae.

All urine samples in our study comprised symptomatic patients visiting the hospital, possibly precluding asymptomatic UTI cases. For a distinct picture, it's important to determine the prevalence and antibiotic sensitivity patterns of bacterial isolates from asymptomatic UTI patients in the same area and link the trends with the current data.

## Conclusions

Our study, despite a low number of study participants and a smaller sample size, draws important conclusions that nitrofurantoin, fosfomycin, and amoxicillin-clavulanate are the best first-line oral empiric antimicrobial drugs in adult patients presenting with urinary tract infections in the AlMajmaah region while amikacin and piperacillin-tazobactam are preferred first-line injectable antimicrobial drugs. Trimethoprim-sulfamethoxazole and ciprofloxacin are no longer recommended as first-line empiric drugs to treat urinary tract infections.
